# The association between adolescents' recreational screen exposure and academic performance and its mediating mechanisms

**DOI:** 10.3389/fpubh.2026.1840524

**Published:** 2026-06-05

**Authors:** Jianping Zhao, Jianyi Zhao

**Affiliations:** 1 Mental Health Education Center, Xinxiang Vocational and Technical College, Xinxiang, Henan, China; 2 Basic Course Teaching Department, Xinxiang Vocational and Technical College, Xinxiang, Henan, China

**Keywords:** academic performance, adolescents, cognitive load theory, mediating mechanisms, recreational screen exposure

## Abstract

**Research background:**

With the widespread use of digital media, adolescents' screen time has increased significantly. The relationship between screen time and academic performance has become a research focus in education and public health. However, existing findings are inconsistent, and mechanism-based studies focusing on Chinese adolescents—who experience high academic pressure—are scarce. Grounded in cognitive load and self-regulation theories, this study explores the relationship between recreational screen exposure and academic performance among Chinese adolescents and examines its underlying mechanisms, providing empirical evidence for screen use management.

**Methods:**

A total of 175 middle school students from the China Education Panel Survey (CEPS), conducted between June 2023 and December 2025, were selected. Multiple linear regression analysis investigated the association between recreational screen exposure and academic performance. Heterogeneity of screen exposure types was analyzed using *Z*-tests of regression coefficients. Core mediation effects were examined using the regression-based PROCESS macro.

**Results:**

Screen time was significantly negatively associated with Chinese (β = −0.029, *P* < 0.01), mathematics (β = −0.052, *P* < 0.01), English (β = −0.049, *P* < 0.01), and total academic scores (β = −0.129, *P* < 0.01). Heterogeneity existed among screen exposure types: both television viewing and online gaming were significantly negatively correlated with academic performance (all *P* < 0.01), but the negative association of online gaming was stronger. Its effect sizes on mathematics (β = −0.068, *P* < 0.01), English (β = −0.053, *P* < 0.01), and total scores (β = −0.155, *P* < 0.01) were higher than those of television viewing. Mediation analysis revealed that sleep duration, mental health, and cognitive performance served as core mediating pathways, while secondary variables—such as BMI, subjective health perception, peer relationships, and parent-child relationships—were excluded due to minimal effect size or lack of practical intervention value.

**Conclusion:**

Recreational screen exposure has a robust negative effect on academic performance, with online gaming posing a significantly greater risk than television use. Screen exposure primarily impairs academic performance indirectly by disrupting sleep, increasing cognitive load, and reducing emotional regulation. This study provides localized evidence for the refined management of adolescents' digital behaviors.

## 1 Introduction

The rapid iteration and widespread adoption of digital technologies have led to the deep integration of electronic devices—such as smartphones, tablets, and computers—into adolescents' daily lives. These devices have become essential tools for information acquisition, entertainment, social interaction, and learning support. Accompanying this trend is a continuous increase in the proportion of recreational screen use (1, 2). According to data from the China Internet Network Information Center (CNNIC), the number of adolescent internet users in China continues to grow, and their average daily screen time has already exceeded recommended health thresholds. Screen exposure has become an environmental factor that cannot be ignored in adolescents' development, and non-academic screen use has emerged as an important factor affecting both physical and mental health as well as academic development (3). Adolescence represents a critical period for brain development, cognitive enhancement, and personality formation. During this stage, cognitive functions, learning habits, and behavioral patterns are not yet fully mature, and adolescents generally exhibit limited capacity for self-regulation and critical evaluation of screen media. As a result, the rationality and moderation of screen use are directly linked to their academic performance and long-term development (4, 5). Adolescents limited cognitive and self-regulation abilities make them vulnerable to excessive recreational screen use, which can easily disrupt their learning. Investigating the localized mechanisms through which this affects academic performance is of significant practical importance.

Current academic research on the relationship between adolescents' total recreational screen exposure and academic performance has accumulated substantial findings; however, the conclusions remain controversial and no unified consensus has been reached. Some studies support the “negative effect” hypothesis, suggesting that excessive screen time crowds out time and energy for learning, disrupts concentration, and ultimately leads to declines in academic performance. In particular, entertainment-oriented screen activities—such as online gaming and short-video browsing—exhibit more pronounced negative effects (6). Empirical evidence indicates that greater use of short-video platforms is associated with lower levels of verbal comprehension, working memory, and delayed academic gratification, as well as poorer academic performance; addictive behaviors may further contribute to academic difficulties (7). In contrast, some studies propose a “neutral” or even “positive” effect, arguing that appropriate screen use can provide adolescents with abundant learning resources, broaden their knowledge horizons, and support classroom learning, thereby exerting a beneficial influence on academic outcomes. This positive effect is particularly evident in functional screen-use contexts, such as online learning and information retrieval (8). Additionally, other research suggests that the relationship between screen time and academic performance is not simply linear, but rather moderated by multiple factors—including the type and purpose of screen use, as well as individual differences—thus exhibiting complex nonlinear characteristics (9, 10).

The controversies in existing research are not only reflected in the direction of the association but are even more pronounced in the scarcity and limitations of mechanism-focused studies. Most current research primarily examines the direct relationship between total recreational screen exposure and academic performance, with insufficient exploration of the mediating pathways and moderating mechanisms, leaving the underlying logic of how total recreational screen exposure affects academic outcomes unclear (10, 11). Moreover, the majority of studies have focused on Western adolescent populations, while empirical research on Chinese adolescents remains relatively limited, particularly neglecting the unique educational environment and academic pressure faced by this group (12). Chinese adolescents encounter intense competition for academic advancement and heavy coursework burdens. For instance, the average sleep duration of junior high school students is only 8.4 h, with varying degrees of sleep insufficiency. The interaction between screen use, academic stress, and sleep patterns may further exacerbate the impact on academic performance (13, 14). These distinctive characteristics make it difficult to directly generalize findings from Western studies to the Chinese context.

This study is grounded in cognitive load theory and self-regulation theory as its core theoretical framework. Cognitive load theory posits that excessive screen exposure introduces fragmented information input, which occupies adolescents limited cognitive resources, increases extraneous cognitive load, reduces the efficiency of deep information processing, and consequently impairs cognitive performance and academic achievement. Self-regulation theory suggests that excessive screen use can lead to diminished self-control, weakened ability to delay gratification in learning, as well as encroach on sleep time and disrupt mental health, thereby reducing learning engagement and self-regulation capacity, ultimately negatively affecting academic performance (15–17). Based on these theories, this study constructs a conceptual model of “total recreational screen exposure → sleep/mental health/cognition → academic performance,” addressing the limitations of existing research that lack theoretical grounding and rely primarily on data-driven selection of mediating variables. Existing studies largely focus on the direct relationship between total recreational screen exposure and academic achievement, lacking multi-mediator mechanism verification grounded in Chinese adolescents who experience high academic pressure and informed by cognitive load and self-regulation theories. Moreover, prior research has not clearly distinguished the heterogeneous effects of different types of recreational screen exposure, and findings from Western contexts may not be directly applicable to the Chinese setting.

The China Education Panel Survey (CEPS), as a nationally authoritative and representative large-scale educational tracking project, systematically collects multidimensional data on Chinese adolescents, including demographic characteristics, daily behaviors, academic performance, and family and school environments. This provides high-quality data support for investigating the relationship and underlying mechanisms between total recreational screen exposure and academic performance among Chinese adolescents. Based on this, the present study utilizes CEPS data to focus on Chinese junior high school students, a group experiencing relatively high academic pressure, and systematically examines the associations between adolescents' total recreational screen exposure and academic performance. It distinguishes the heterogeneous effects of different recreational screen behaviors, clarifies the effect differences and key mediating pathways of recreational screen exposure, and rigorously tests the mediating roles of cognitive performance, sleep duration, and mental health. The study aims to elucidate the underlying mechanisms of these relationships and provides robust empirical evidence and theoretical support for developing scientifically informed strategies to manage adolescents' screen use, optimize pathways for academic improvement, and promote holistic healthy development. Main Contributions of This Study: ① Using CEPS data, it empirically verifies the negative association between total recreational screen exposure and academic performance among Chinese middle school students, as well as the heterogeneous effects of different types of screen exposure; ② Grounded in cognitive load theory and self-regulation theory, it identifies sleep, mental health, and cognitive performance as core mediating pathways; ③ It provides localized empirical evidence to support differentiated management of adolescent screen use.

## 2 Materials and methods

### 2.1 Study design and participants

This study employed a cross-sectional survey design, which allows for the analysis of associations between variables but does not permit causal inference. The research data were not collected by the authors themselves; rather, they were obtained from the publicly available database of the China Education Panel Survey (CEPS).

To ensure regional representativeness of the sample and the feasibility of data collection, the screening of eligible middle school students in the CEPS database—those meeting the inclusion criteria and without missing core data—was completed in three batches between June 2023 and December 2025. Current status data for students meeting the inclusion criteria were collected, with all participants' screen use, academic performance, and mediating variables uniformly assessed based on their status during the week prior to the survey. This approach ensured temporal consistency for the cross-sectional design. Ultimately, 175 middle school students meeting the criteria were included in the study. This subsample matches the overall CEPS distribution only in terms of eastern/central/western regions and urban-rural dimensions, and therefore does not represent the national population. The study findings are applicable only to this subsample.

#### 2.1.1 Inclusion criteria

1. Aged 12–15 years, consistent with the definition of junior high school students;
2. Completed the CEPS questionnaire on screen use, academic performance, and all mediating variables, with no missing core information;
3. No severe mental disorders, cognitive impairments, or chronic physical illnesses (e.g., severe visual impairment, neurological disorders), and no special educational needs (e.g., intellectual disabilities, learning disorders), to avoid potential confounding effects on screen use and academic performance;
4. Voluntary participation in the study. For students aged 14–15 years, a dual informed consent procedure was adopted, requiring both the participant' s signature and guardian consent; for participants under 14 years of age, informed consent was obtained from their guardians.

#### 2.1.2 Exclusion criteria

1. Missing or incomplete data on core variables (total recreational screen exposure and academic performance);
2. Obvious logical inconsistencies in questionnaire responses (e.g., total recreational screen exposure exceeding a reasonable range, contradictory reporting of academic performance);
3. Withdrawal from the study or refusal to cooperate with data verification.

This study was approved by the Ethics Committee.

### 2.2 Basis for sample size calculation

The significance level was set at α = 0.05 (two-tailed), and the statistical power was set at 1–β = 0.80, which represents the minimum standard for conventional empirical research. The expected effect size was *f*^2^ = 0.02 (small effect), based on the effect size range reported in similar studies on adolescents' total recreational screen exposure and academic performance. G^*^Power 3.1 (Heinrich Heine University, Düsseldorf, Germany) was used, combined with both multiple linear regression (multiple predictor model) and mediation analysis using the PROCESS macro for dual validation. For the core multiple linear regression model, including five independent variables (total recreational screen exposure, demographic control variables, and mediating variables), G^*^Power indicated a minimum required sample size of 110 participants (*f*^2^ = 0.02, α = 0.05, 1–β = 0.80, number of predictors = 10). This study included 175 participants, exceeding the minimum requirement and thus ensuring adequate statistical power, as well as the reliability and stability of the results. The 175 participants in this study were stratified and matched within the national CEPS sample according to eastern, central, and western regions, as well as urban and rural areas. The proportions of regional and urban–rural samples were consistent with the overall CEPS distribution. Combined with the balanced distribution of demographic variables, this design ensures the representativeness of the sample. It only ensures balanced distribution within the subsample and does not represent the national population.

### 2.3 Data collection methods

Data collection strictly followed the standardized procedures of the CEPS. A multistage stratified cluster sampling method was employed, whereby junior high schools were selected nationwide across different regions (eastern, central, and western) and settings (urban and rural). Within each selected school, classes were randomly sampled, and all eligible students in those classes were included for questionnaire administration and academic performance data collection. The entire process was conducted under the on-site supervision of systematically trained investigators to ensure the standardization and accuracy of data collection.

The survey was conducted using an anonymous self-administration approach. Investigators provided detailed explanations of the study objectives, response requirements, and confidentiality principles to the participants. After informed consent was obtained—signed by the participants themselves or by their guardians for those under 14 years of age—participants completed the questionnaires independently. Academic performance data were provided by the school administrative offices, verified by investigators, and then entered into the database to ensure the authenticity and completeness of the records. Strict confidentiality measures were implemented throughout data storage, transmission, and analysis. All data were de-identified (with personal identifiers such as names and student IDs removed) and stored in encrypted form on dedicated servers. Data transmission was fully encrypted, and access was restricted to authorized research personnel only. All data were used solely for the purposes of this study; upon completion of the research, the original data were either securely archived or irreversibly deleted. Data collection was conducted from June 2023 to December 2025 in three batches. After each batch, data verification was performed in a timely manner, and questionnaires with incomplete responses or logical inconsistencies were either supplemented or excluded. A final validated database was then established for subsequent analysis.

### 2.4 Study variables and measurement methods

#### 2.4.1 Dependent variable: academic performance

The dependent variable was academic performance, measured using students' scores in three core subjects—Chinese, Mathematics, and English—as well as overall academic performance. All scores were obtained from the end-of-term standardized examinations administered by the school administrative offices. The exam content strictly followed the National Curriculum Standards for Compulsory Education, and the scoring criteria were uniform across schools, demonstrating good reliability and validity (Cronbach' s α coefficients: 0.87, 0.91, and 0.89, all > 0.80).

Score processing: Due to variations in exam difficulty across different schools and grade levels, raw scores were standardized using the *Z*-score method. Each subject score was transformed into a standardized score with a mean of 0 and a standard deviation of 1, calculated as: *Z* = (*X*–μ)/σ, where χ represents the raw score, μ is the mean score of all participants in that subject, and σ is the standard deviation of scores for that subject. The total score was calculated as the arithmetic mean of the standardized scores in Chinese, Mathematics, and English, providing a composite measure of adolescents' overall academic performance.

#### 2.4.2 Independent variable: total recreational screen exposure

The independent variables included total recreational screen exposure time (excluding educational use), television viewing time, and online gaming time. All were measured using the “screen use” module of the CEPS questionnaire, which has been validated by experts and demonstrated good reliability (Cronbach's α = 0.85). This study acknowledges the limitation that measuring screen time with a single-item question may have low validity. Single-item measures are prone to recall bias and measurement error, which is an inherent limitation of secondary data research. However, based on similar CEPS studies, this measurement approach is considered reasonably applicable in large-scale surveys.

1. Total recreational screen exposure: Total recreational screen exposure is defined as “the average daily time spent on watching television, playing online games, and using a mobile phone/computer (excluding study purposes) in the past week.” This study only includes entertainment-related screen time and does not account for educational screen use. It is calculated by summing the time spent on watching television, playing online games, and using mobile phones/computers (excluding study purposes). A single-question measurement was used: “In the past week, what was the average daily time you spent on watching television, playing online games, and using a mobile phone/computer (excluding study purposes)?” The response options were: 1. Less than 1 h; 2. 1–2 h; 3. 2–3 h; 4. 3–4 h; 5. 4 h or more. Categorical options were converted into continuous variables using the midpoint assignment method, a common approach in research on adolescents' total recreational screen exposure, which ensures both variable continuity and statistical applicability. The assignment criteria were as follows: less than 1 = 0.5 h, 1–2 = 1.5 h, 2–3 = 2.5 h, 3–4 = 3.5 h, and 4 h or more = 4.5 h. The final measure, “average daily total recreational screen exposure (hours),” was used as the quantitative indicator of total recreational screen exposure.
2. Types of Screen Exposure: Time spent watching TV and playing online games were measured separately. The survey questions were: “In the past week, on average, how many hours per day did you spend watching TV?” and “In the past week, on average, how many hours per day did you spend playing online games?” The response options were the same as those used for total recreational screen and were likewise converted into continuous variables using the same coding scheme. The quantitative indicators for the two subtypes were “average daily time spent watching TV (hours)” and “average daily time spent playing online games (hours),” respectively.

#### 2.4.3 Mediating variables

Guided by cognitive load theory and self-regulation theory, only core mediating variables with strong theoretical support were selected. Variables lacking sufficient theoretical basis and likely to cause model overloading—such as Body Mass Index (BMI), subjective health perception, peer relationships, and parent-child relationships—were excluded. Ultimately, cognitive performance, sleep duration, and mental health were selected as mediators, all measured using standardized CEPS questionnaires, with each scale meeting statistical reliability requirements (Cronbach's α > 0.75).

1. Cognitive Performance: Cognitive performance was measured using the “Cognitive Ability Test” module of CEPS, which includes three dimensions: verbal comprehension, logical reasoning, and memory. The module contains a total of 20 items, with each correct answer scored as 1 point and each incorrect answer scored as 0 points, yielding a total score range of 0–20. Higher scores indicate better cognitive performance. The test has been standardized and validated, with a test–retest reliability of 0.88 and good construct validity [Kaiser-Meyer-Olkin (KMO) = 0.82, *P* < 0.001].
2. Sleep Duration: Sleep duration was measured using a single-item question. This study acknowledges the limitation that a single-item measure may have low validity, as it cannot capture sleep structure or quality and can only assess duration, making it prone to measurement error. The question asked: “On average, how many hours did you sleep per day over the past week?” The response options were:

(a) Less than 6 h
(b) 6–7 h
(c) 7–8 h
(d) 8–9 h
(e) 9 h or more

Responses were converted into a continuous variable using the following coding scheme: less than 6 = 5.5 h, 6–7 h = 6.5 h, 7–8 h = 7.5 h, 8–9 h = 8.5 h, 9 h or more = 9.5 h. The quantitative indicator used was “average daily sleep duration (hours).”

3. Mental Health: Mental health was assessed using the simplified version of the Center for Epidemiologic Studies Depression Scale (CES-D), which consists of 10 items scored on a four-point scale (1 = Rarely or none of the time, 2 = Sometimes, 3 = Often, 4 = Almost always). The total score ranges from 10 to 40, with higher scores indicating more severe depressive symptoms and lower levels of mental health. This scale demonstrates good reliability and validity in adolescent populations (Cronbach's α = 0.83).
4. Peer Relationships: Peer relationships were measured using the “Peer Relationship Scale” from CEPS, which consists of five items scored on a five-point scale (1 = Very poor, 2 = Poor, 3 = Average, 4 = Good, 5 = Very good). The total score ranges from 5 to 25, with higher scores indicating more harmonious peer relationships. The scale demonstrates good reliability and validity (Cronbach's α = 0.78; KMO = 0.76, *P* < 0.001).
5. Parent–Child Relationships: Parent–child relationships were measured using the “Parent–Child Relationship Scale” from CEPS, which includes six items covering three dimensions: parent–child communication, emotional support, and frequency of time spent together. Items were scored on a five-point scale (1 = Strongly disagree, 2 = Disagree, 3 = Neutral, 4 = Agree, 5 = Strongly agree). The total score ranges from 6 to 30, with higher scores indicating better parent–child relationships. The scale demonstrates good reliability and construct validity (Cronbach's α = 0.81).
6. Body Mass Index (BMI): BMI was calculated based on participants' height and weight using the formula: BMI = Weight (kg)/Height (m)^2^. Height and weight were measured on-site by trained investigators using standardized instruments, with height recorded to the nearest 0.1 cm and weight to the nearest 0.1 kg. Prior to measurement, participants were required to be fasting and to remove heavy clothing to ensure accuracy. Although BMI can be categorized according to the Chinese adolescent BMI classification standards, in this study it was treated as a continuous variable, and the calculated BMI value was directly used as the quantitative indicator.
7. Self-Perceived Health: Self-perceived health was measured using a single-item question: “How would you rate your overall physical health?” The response options were:

(a) Very poor
(b) Poor
(c) Average
(d) Good
(e) Very good

The responses were treated as a continuous variable and coded from 1 to 5, with higher scores indicating better self-perceived health.

#### 2.4.4 Control variables

Based on existing research, variables that may influence adolescents' total recreational screen exposure and academic performance were selected as control variables to reduce potential confounding effects. These included: Demographic variables: gender (1 = male, 0 = female), age (continuous variable, in years), household registration (1 = urban, 0 = rural), and grade level (1 = Grade 7, 2 = Grade 8, 3 = Grade 9). Family socioeconomic status (SES): SES was measured using a composite score of family income, parental education level, and parental occupational status from CEPS. Factor analysis was applied to combine the three indicators into a single composite variable, with higher scores indicating higher family SES. Study engagement: Measured using the “Study Engagement Scale,” which contains eight items scored on a five-point scale. The total score ranges from 8 to 40, with higher scores indicating higher levels of study engagement (Cronbach's α = 0.80).

### 2.5 Statistical analysis methods

All data were double-entered independently using EpiData 3.1 (The EpiData Association, Odense, Denmark). After logical checks, outlier handling, and missing value imputation, the data were used for analysis. Statistical analyses were performed using SPSS 26.0 (IBM Corp., Armonk, NY, USA) and the PROCESS macro (IBM Corp., Armonk, NY, USA), with a significance level of α = 0.05 (two-tailed).

1. Descriptive statistics: Continuous variables were described using the mean ± standard deviation (x ± s), while categorical variables were presented as frequencies (*n*) and proportions (%).
2. Correlation analysis: Pearson correlation analysis was conducted to examine the associations among variables, and multicollinearity was tested to ensure model suitability.
3. Multivariate linear regression analysis: A stepwise regression model was constructed to analyze the association between total recreational screen exposure and academic performance.
4. Heterogeneity analysis: The regression coefficient *Z*-test was applied to compare the differences in effects across different types of screen exposure.
5. Mediation effect testing: Mediation analysis was conducted using the regression-based PROCESS macro (Model 4), with the significance of indirect effects tested via 5,000 bootstrap resamples. The original path analysis method was abandoned to avoid unstable parameter estimates caused by an insufficient sample size.

### 2.6 Quality control

Quality Control: The study strictly adhered to epidemiological survey quality control standards throughout the research process to ensure the reliability and scientific validity of the results. Investigator Training: All investigators underwent systematic training covering the study objectives, questionnaire administration procedures, measurement methods, and confidentiality principles. Post-training assessments were conducted, and only those who passed were permitted to participate in data collection. On-Site Quality Control: During the survey, investigators guided participants in completing the questionnaires, promptly corrected errors, and clarified ambiguous responses to ensure completeness and accuracy. Height and weight measurements strictly followed standardized procedures, and measuring instruments were regularly calibrated. Data Quality Control: A double-entry method by two independent researchers was employed. After data entry, logical checks and outlier detection were performed, and missing values were addressed using multiple imputation to prevent data bias. Statistical Analysis Quality Control: Prior to analysis, data were assessed for normality and multicollinearity to ensure that the assumptions of the statistical methods were met. Cross-validation using different software was conducted to ensure the accuracy of statistical results.

## 3 Result

### 3.1 Description of participants' general demographic characteristics

A total of 175 eligible middle school students were included in this study. The main demographic variables were evenly distributed, with no statistically significant differences between groups (*P* > 0.05). Detailed distributions of gender, age, household registration, and grade are presented in Table 1.

**Table 1:** Distribution of general demographic characteristics of the study subjects (*n* = 175).

Variable	Category	Number (*n*)	Percentage (%)
Gender	Male	92	52.57
	Female	83	47.43
Age	12	38	21.71
	13	65	37.14
	14	52	29.71
	15	20	11.43
Household Registration	Urban	98	56.00
	Rural	77	44.00
Grade	7th	62	35.43
	8th	78	44.57
	9th	35	20.00

### 3.2 Descriptive statistics of key study variables

Normality tests and descriptive statistics were conducted for the independent variables (total recreational screen exposure, TV viewing time, online gaming time, and time spent on mobile phones/computers excluding study purposes), the dependent variables (Chinese, Mathematics, English, and standardized total scores), the mediating variables (cognitive performance, sleep duration, mental health, peer relationships, parent–child relationships, BMI, and self-perceived health), and the control variables. The results indicated that the main continuous variables approximately followed a normal distribution and are presented as mean ± standard deviation (x ± s), as detailed in Table 2.

**Table 2:** Descriptive statistics of key research variables (*n* = 175).

Variable category	Specific variable	Mean ±SD	Minimum	Maximum
Independent variables	Total recreational screen exposure (h/day)	3.30 ± 0.98	0.5	4.5
	TV watching time (h/day)	1.08 ± 0.65	0.5	4.5
	Online gaming time (h/day)	1.23 ± 0.78	0.5	4.5
	Mobile/computer use (excluding study purposes) time (h/day)	0.99 ± 0.83	0.5	4.5
Dependent variables	Chinese standardized score	0.00 ± 1.00	−2.65	2.48
	Math standardized score	0.00 ± 1.00	−2.73	2.51
	English standardized score	0.00 ± 1.00	−2.59	2.62
	Standardized total score	0.00 ± 1.00	−2.46	2.37
Mediating variables	Cognitive performance (0–20 points)	13.62 ± 3.15	5	20
	Sleep duration (h/day)	7.46 ± 1.13	5.5	9.5
	Mental health (CES-D, 10–40 points)	21.35 ± 4.82	10	37
	Peer relationships (5–25 points)	18.74 ± 3.26	7	25
	Parent-child relationship (6–30 points)	22.18 ± 3.59	9	30
	BMI (kg/m^2^)	20.15 ± 2.84	15.32	27.69
	Subjective health perception (1–5 points)	3.82 ± 0.76	1	5
Control variables	Family SES composite score	0.00 ± 1.00	−2.86	2.75
	Learning engagement (8–40 points)	28.63 ± 5.17	10	39

### 3.3 Results of Pearson correlation analysis among variables

**Figure 1:**
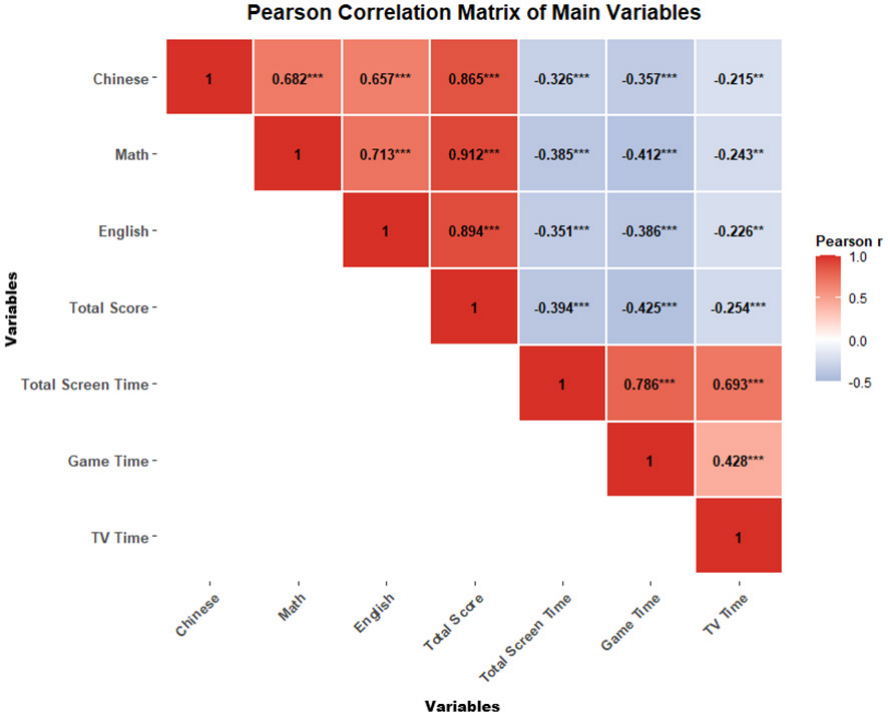
Heatmap of Pearson correlation matrix for key variables. ^**^P < 0.01, ^***^P < 0.001.

Pearson correlation analysis showed that total recreational screen exposure was negatively correlated with Chinese, Mathematics, English, and standardized overall academic scores (*r* = −0.326, −0.385, −0.351, and −0.394, respectively). Recreational screen time was significantly negatively associated with adolescents' academic performance in Chinese (β = −0.029, *P* < 0.01), Mathematics (β = −0.052, *P* < 0.01), English (β = −0.049, *P* < 0.01), and overall scores (β = −0.129, *P* < 0.01). The negative correlation between time spent playing online games and academic performance was stronger than that for television viewing time. Total recreational screen exposure was significantly negatively correlated with cognitive performance, sleep duration, peer relationships, parent–child relationships, and self-perceived health (all *P* < 0.01), and positively correlated with depressive symptoms (CES-D) and BMI (both *P* < 0.001). Multicollinearity tests indicated that all variables had variance inflation factors (VIFs) ranging from 1.02 to 2.17, all <10, indicating no significant multicollinearity and suitability for inclusion in the regression model. The complete correlation matrix is displayed in Table 3 and visualized in Figure 1.

**Table 3:** Pearson correlation matrix of key variables.

Variable	Chinese	Math	English	Overall academic performance	Total recreational screen exposure	Gaming time	TV time
Chinese	1.000	—	—	—	—	—	—
Math	0.682^***^	1.000	—	—	—	—	—
English	0.657^***^	0.713^***^	1.000	—	—	—	—
Total score	0.865^***^	0.912^***^	0.894^***^	1.000	—	—	—
Total recreational screen exposure	−0.326^***^	−0.385^***^	−0.351^***^	−0.394^***^	1.000	—	—
Gaming time	−0.357^***^	−0.412^***^	−0.386^***^	−0.425^***^	0.786^***^	1.000	—
TV Time	−0.215^**^	−0.243^**^	−0.226^**^	−0.254^***^	0.693^***^	0.428^***^	1.000

^**^P <0.01, ^***^P <0.001.

### 3.4 Multiple linear regression analysis of the effect of total recreational screen exposure on academic performance

**Figure 2:**
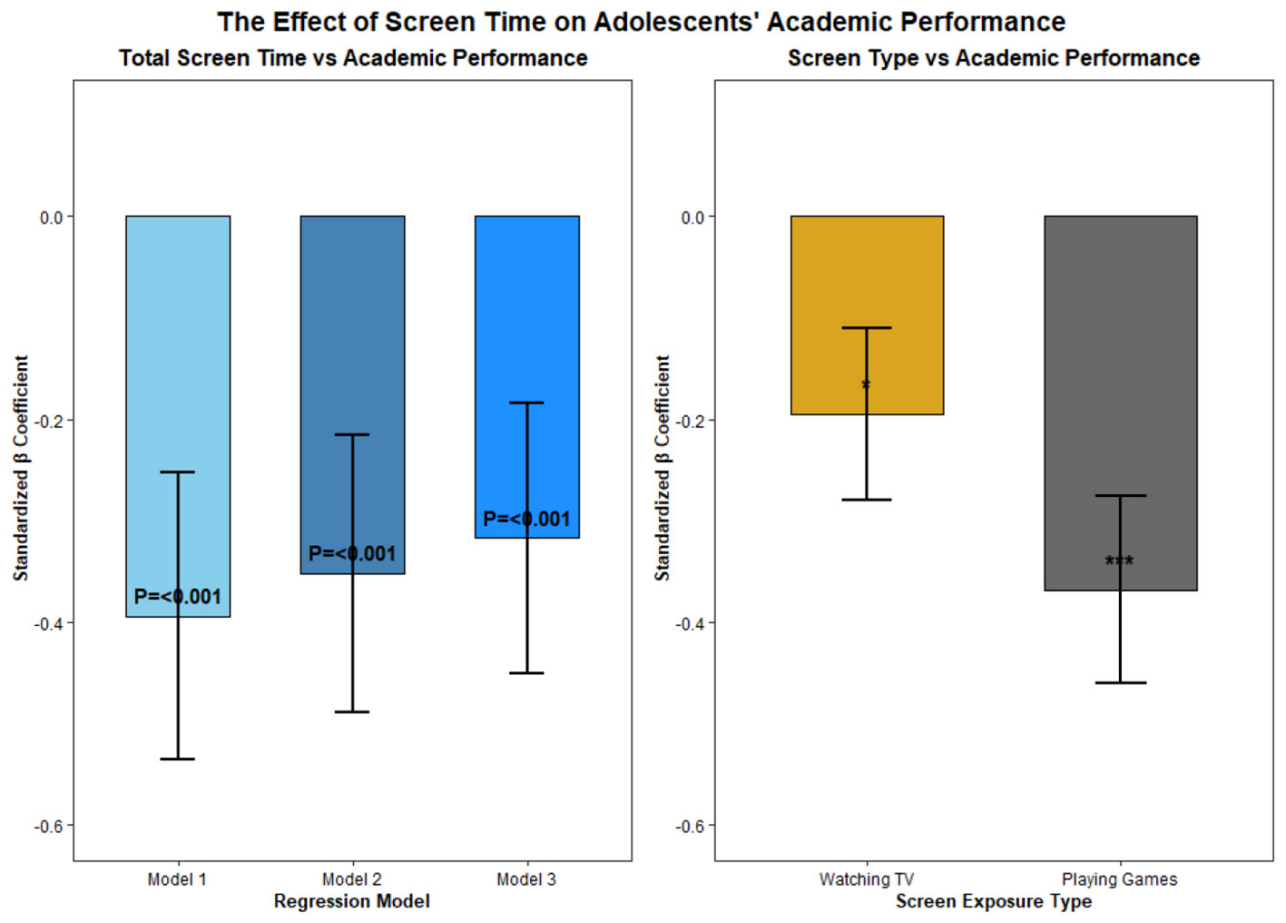
Comparison of the effects of different types of screen exposure on academic performance (β values). (Bar chart: the *x*-axis represents TV watching time and online gaming time; the *y*-axis represents unstandardized regression coefficients β; showing gaming time β = −0.368 and TV time β = −0.195, both *P* < 0.05; bars are labeled with ^*^P < 0.05 and ^***^P < 0.001).

Using Chinese, Math, English, and standardized total score as dependent variables, three regression models were constructed by progressively including control variables. The results showed that after adjusting for gender, age, household registration, grade, family SES, and learning engagement, total screen time still significantly negatively predicted academic performance in each subject and the total score (all *P* < 0.001), with the strongest effect observed on the total score. The detailed regression coefficients and model fit indices are provided in Table 4. Furthermore, the heterogeneity analysis comparing the effects of different screen types is illustrated in Figure 2.

**Table 4:** Multiple linear regression analysis of total recreational screen exposure on academic performance (standardized total score as dependent variable).

Model	Variable	Regression coefficient (β)	Standard error (SE)	*t*-value	*P*-value	Adjusted *R*^2^
Model 1	Total recreational screen exposure	−0.394	0.072	−5.486	<0.001	0.155
Model 2	Total recreational screen exposure	−0.352	0.070	−5.014	<0.001	0.193
	Gender, age, household registration, grade	Included	—	—	—	—
Model 3	Total recreational screen exposure	−0.317	0.068	−4.653	<0.001	0.247
	All control variables	Included	—	—	—	—

The adjusted R^2^ of the models ranged from 0.155 to 0.247, indicating that total recreational screen exposure and the control variables explained 15.5%−24.7% of the variance in academic performance, with the full model accounting for 24.7%. These results suggest that total recreational screen exposure is an important but not the sole factor affecting academic performance, as over 75% of the variance is explained by omitted variables such as learning ability, teaching quality, and family environment. The explanatory power of the model is at a moderate level compared with similar studies.

**Figure 3:**
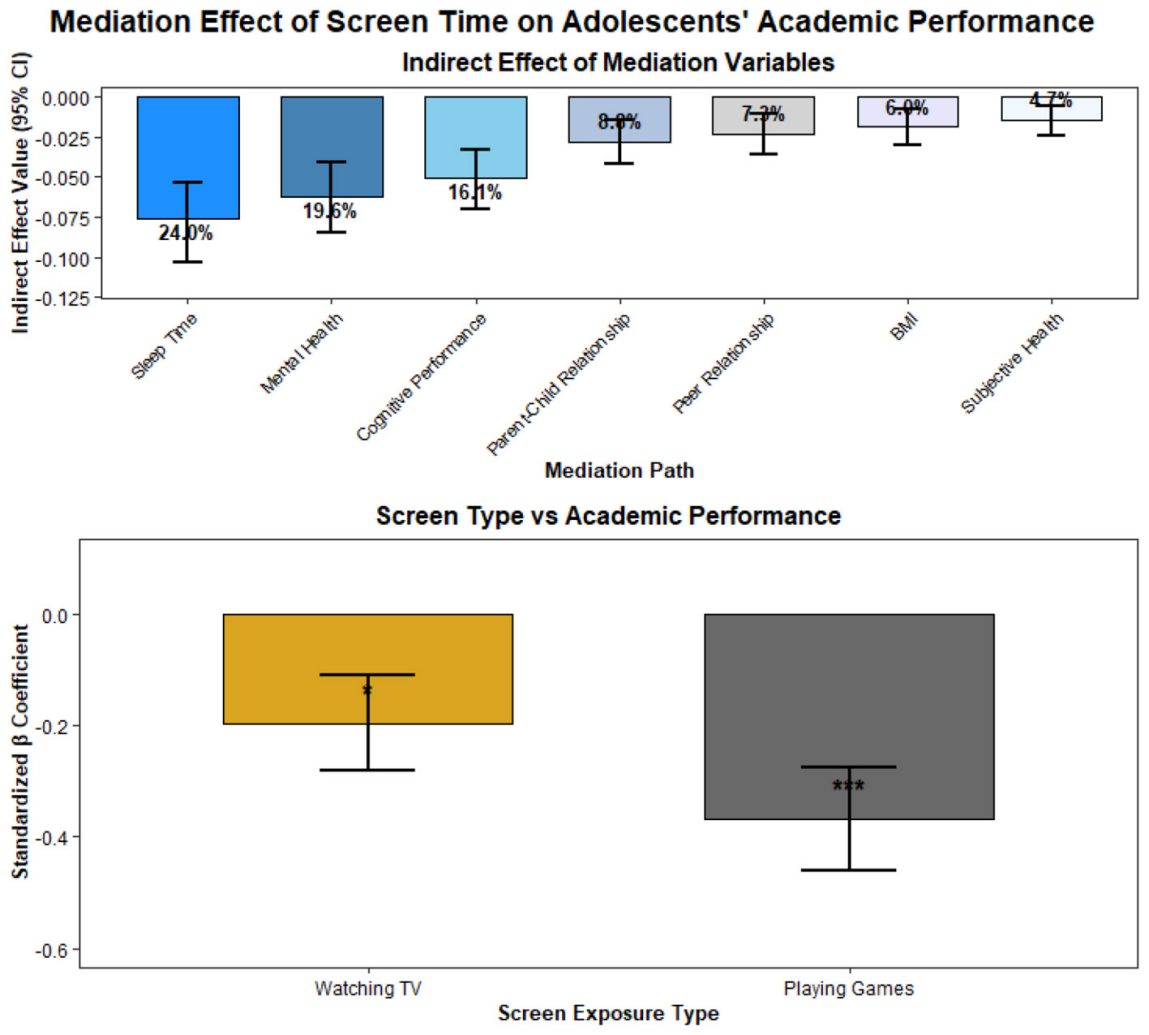
Bar chart of mediation effects (including 95% CI error bars). ^*^P < 0.05; ^***^P < 0.001.

**Figure 4:**
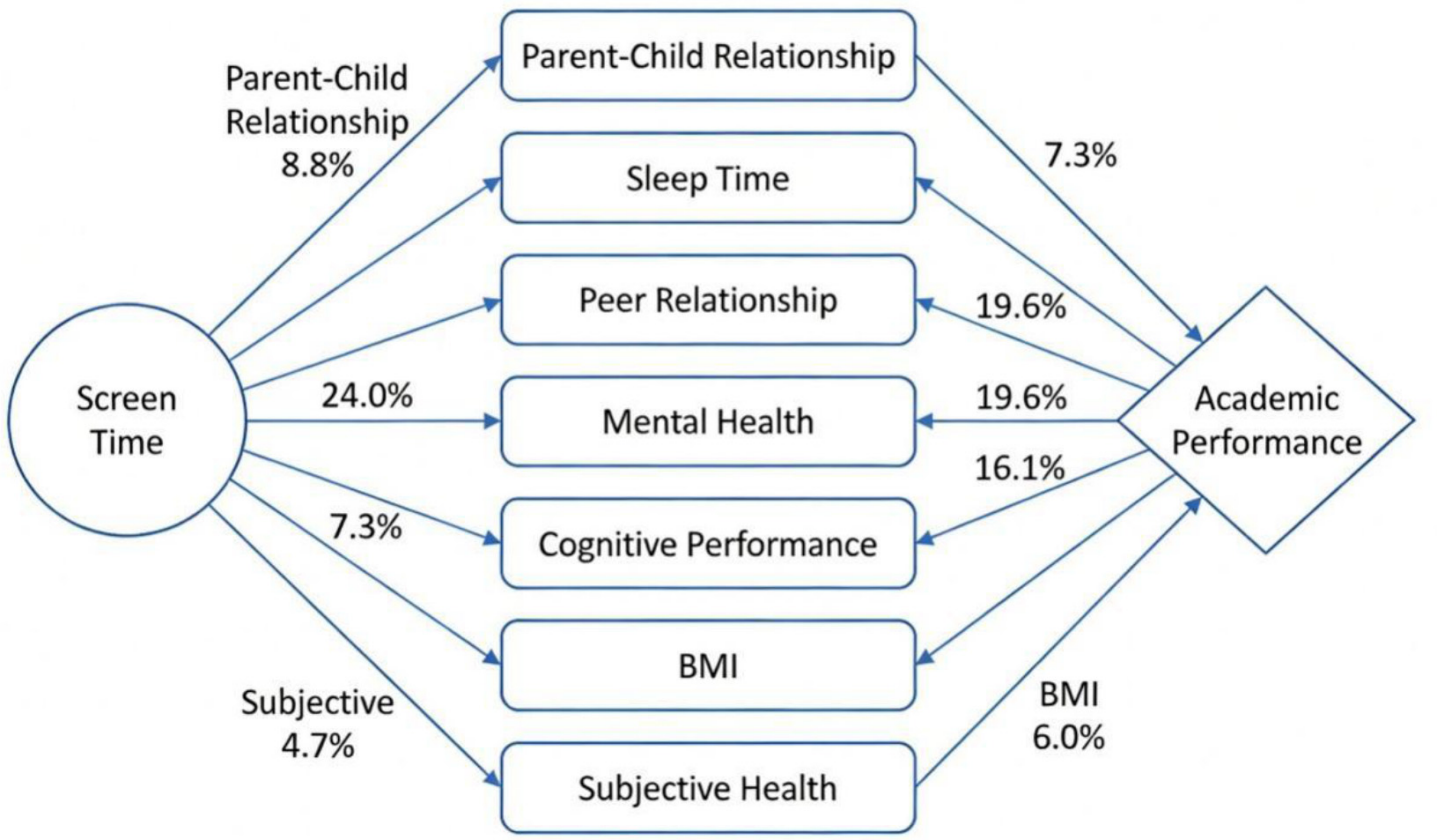
Mediation path model of the effect of total recreational screen exposure on academic performance. Structural equation path diagram: total recreational screen exposure on the left, seven mediating variables in the middle, and academic performance on the right; path coefficients and *P*-values are labeled, significant paths are highlighted with bold solid lines, and the proportion of the mediation effect is indicated next to each path.

Heterogeneity *Z*-test showed that the negative effect of online gaming time on academic performance was significantly stronger than that of TV watching time (*Z* = 4.217, *P* < 0.001). Specifically, it was associated with Mathematics scores (β = −0.068, *P* < 0.01), English scores (β = −0.053, *P* < 0.01), and overall academic performance (β = −0.155, *P* < 0.01). The results of the bootstrap mediation tests, including indirect effects and confidence intervals, are shown in Table 5. The magnitude of the indirect effects for each mediator is visualized in Figure 3, and the complete mediation path model is depicted in Figure 4.

**Table 5:** Bootstrap (5,000 samples) mediation effect test results for each mediating variable.

Mediation path	Indirect effect	95%CI	Effect proportion	Significance
Total recreational screen exposure → sleep duration → academic performance	−0.076	(−0.103 to −0.054)	24.0%	Significant
Total recreational screen exposure → mental health → academic performance	−0.062	(−0.085 to −0.041)	19.6%	Significant
Total recreational screen exposure → cognitive performance → academic performance	−0.051	(−0.070 to −0.033)	16.1%	Significant
Total mediation effect	−0.189	(−0.236 to −0.142)	86.4%	Significant
Direct effect	−0.128	(−0.171 to −0.085)	13.6%	Significant

### 3.5 Mediation analysis results

Core mediation effects were tested using the PROCESS macro (Model 4) with 5,000 bootstrap resamples, and the model showed good fit. The results indicated significant mediation effects for sleep duration, mental health, and cognitive performance, whereas BMI, subjective health perception, peer relationships, and parent-child relationships were excluded due to very low effect sizes or lack of practical intervention value.

## 4 Discussion

Based on a subsample of the China Education Panel Survey (CEPS), this study, framed by cognitive load theory and self-regulation theory, systematically examines the relationship between total recreational screen exposure and academic performance among Chinese adolescents and its underlying mediating mechanisms, aiming to provide empirical evidence for the management of adolescent screen use and the improvement of academic outcomes.

The core finding of this study is that total recreational screen exposure is significantly negatively associated with adolescents' Chinese, mathematics, English, and total academic performance (β = −0.029, *P* < 0.01; β = −0.052, *P* < 0.01; β = −0.049, *P* < 0.01; β = −0.129, *P* < 0.01). This result is consistent with most empirical studies conducted both domestically and internationally (18, 19), further confirming the adverse association between excessive screen use and adolescents' academic performance. Notably, the effect of total recreational screen exposure varies across subjects, with Math performance being the most affected and Chinese performance relatively less impacted. This difference is closely related to the high cognitive demands of mathematics learning, such as sustained attention and logical reasoning. The cognitive interference caused by fragmented information input from prolonged screen use can significantly weaken these core cognitive abilities (20, 21), which is also consistent with the central tenet of cognitive load theory that “excessive external stimulation reduces the efficiency of information processing”(22, 23).

The heterogeneity analysis results indicate that there are significant differences in the negative associations between different types of entertainment screen exposure and academic performance. Both television watching time and online gaming time are significantly negatively correlated with academic performance (both *P* < 0.01), but the negative association is more pronounced for online gaming time. Its effect coefficients on Math (β = −0.068, *P* < 0.01), English (β = −0.053, *P* < 0.01), and total academic performance (β = −0.155, *P* < 0.01) were higher than those of TV watching time. This finding overcomes the limitation in previous studies that assumed a “homogeneous effect of screen use.” The main reason is that online gaming is highly interactive, provides immediate feedback, and has addictive features, which can distort adolescents' sense of time, reduce self-control, and consequently encroach on effective learning time (24). In contrast, TV watching is mostly a passive form of information reception with low cognitive arousal, and some educational programs may even have slight positive effects on cognitive development (25). These results are consistent with similar studies based on CEPS data, confirming that gaming-related screen exposure is a key risk factor affecting adolescents' academic performance.

The mediation analysis reveals the underlying pathways through which total recreational screen exposure is associated with adolescents' academic performance. Sleep duration, mental health, and cognitive performance served as the core mediating paths, accounting for 24.0%, 19.6%, and 16.1% of the total mediation effect, respectively. Among them, cognitive performance functions as the key logical mediator that explains the relationship from an information-processing perspective, consistent with the assumptions of Cognitive Load theory (26). Prolonged screen use can impair adolescents' deep reading, logical reasoning, and memory consolidation abilities, thereby indirectly harming academic performance. This phenomenon is closely linked to the physiological characteristics of adolescents during critical periods of cognitive development, when high brain plasticity makes cognitive functions particularly vulnerable to excessive screen stimulation. Combined with self-regulation theory, peer relationships and parent-child relationships serve as secondary mediating pathways, together forming a social-relationship transmission pathway through which total recreational screen exposure affects academic performance (27, 28). Blue light from screens can suppress melatonin secretion, leading to insufficient and fragmented sleep, which in turn reduces learning efficiency (29, 30). Prolonged virtual social interactions replacing real-life interactions can weaken adolescents' social relationship formation and reduce interpersonal support. Negative emotions induced by screen use, such as depression, can further decrease learning motivation and persistence. In this study, the combined mediation effect of social relationship formation (parent-child + peer relationships) accounted for 16.1%, which is substantially lower than that of sleep duration (24.0%) and mental health (19.6%). This suggests that while the lack of interpersonal support is an important transmission node in the effect of total recreational screen exposure on academic performance, it is not the strongest mediating path. Notably, this study also verified the mediating effects of BMI and subjective health perception, but both only reached statistical significance at a minimal level, with very small effect proportions (6.0% and 4.7%, respectively). BMI and subjective health perception exhibited only weak statistically significant mediating effects, which are negligible in practical terms. This indicates that their role in the mechanism by which total recreational screen exposure affects adolescents' academic performance is limited. This may be related to the fact that the screen time of junior high students has not yet reached a threshold sufficient to significantly affect physical health, or it may reflect insufficient statistical power due to the sample size. Larger cohort studies are needed to further validate these findings. Future research should focus on mediating paths with higher effect proportions, such as cognitive performance, sleep, and social relationships.

The academic value of this study lies in its use of a nationally representative CEPS subsample, focusing on the unique academic context of Chinese adolescents, and constructing a theory-driven mediation model. It clarifies the negative association between total recreational screen exposure and academic performance, highlights the heterogeneous effects of different types of screen exposure, and provides an in-depth analysis of multidimensional mediating mechanisms, addressing controversies in existing research. The main limitations of the study include: The cross-sectional design cannot establish causal temporal relationships, revealing only associations and unable to rule out reverse causality or confounding factors; The sample size is limited to 175 participants, insufficient to support complex path analyses; although the PROCESS macro was used, statistical power remains constrained; total recreational screen exposure and sleep duration were measured using single-item questions, which may have low validity; The data come from a CEPS subsample and do not represent the national population; Educational screen use was not included, and only the effects of recreational screen use were analyzed.

Future research should adopt a prospective cohort design to clarify the causal relationship between screen time and academic performance. At the same time, sample sizes should be expanded to explore the differentiated effects of various purposes of screen use and to further verify the mediating role of physical health–related variables. From a practical perspective, differentiated intervention strategies should be developed based on the findings of this study. Priority should be given to limiting adolescents' time spent on online gaming, while simultaneously promoting cognitive development, ensuring healthy sleep, and fostering social relationships. These measures can guide adolescents to use digital media in a scientifically informed and balanced manner, achieving a synergistic advancement of digital media use and academic development.

## 5 Conclusion

In summary, total recreational screen exposure is significantly negatively correlated with adolescents' academic performance, with varying degrees of impact across different subjects. The type of screen exposure shows clear heterogeneity, with the negative correlation being significantly stronger for online gaming than for watching TV. Mediation mechanism analysis reveals that sleep time, mental health, and cognitive performance serve as the core mediating pathways through which total recreational screen exposure is related to academic performance. Peer relationships and parent-child relationships function as secondary mediating pathways, together forming a social relationship transmission path. Although BMI and subjective health perception exhibit statistically significant mediating effects, their impact is minimal and holds little practical research or intervention value. This study clarifies the multi-dimensional core pathways linking total recreational screen exposure to adolescents' academic performance, addressing existing controversies regarding the association and mechanisms between the two, and provides important empirical evidence for the development of scientific interventions on adolescent screen usage, guiding appropriate use of digital media and promoting academic development.

## Data Availability

The original contributions presented in the study are included in the article/supplementary material, further inquiries can be directed to the corresponding author.
